# Metabolic and amyloid PET network reorganization in Alzheimer’s disease: differential patterns and partial volume effects

**DOI:** 10.1007/s11682-019-00247-9

**Published:** 2020-03-03

**Authors:** Gabriel Gonzalez-Escamilla, Isabelle Miederer, Michel J. Grothe, Mathias Schreckenberger, Muthuraman Muthuraman, Sergiu Groppa, for the Alzheimer’s Disease Neuroimaging Initiative

**Affiliations:** 1 Department of Neurology, Focus Program Translational Neuroscience (FTN), Rhine Main Neuroscience Network (rmn2), University Medical Center of the Johannes Gutenberg University Mainz, Langenbeckstr. 1, 55131 Mainz, Germany; 2 Department of Nuclear Medicine, University Medical Center of the Johannes Gutenberg University Mainz, Mainz, Germany; 3 German Center for Neurodegenerative Diseases (DZNE) - Rostock/Greifswald, Rostock, Germany

**Keywords:** Alzheimer’s disease, Brain molecular networks, MRI, Partial volume effects correction, PET

## Abstract

Alzheimer’s disease (AD) is a neurodegenerative disorder, considered a disconnection syndrome with regional molecular pattern abnormalities quantifiable by the aid of PET imaging. Solutions for accurate quantification of network dysfunction are scarce. We evaluate the extent to which PET molecular markers reflect quantifiable network metrics derived through the graph theory framework and how partial volume effects (PVE)-correction (PVEc) affects these PET-derived metrics 75 AD patients and 126 cognitively normal older subjects (CN). Therefore our goal is twofold: 1) to evaluate the differential patterns of [^18^F]FDG- and [^18^F]AV45-PET data to depict AD pathology; and ii) to analyse the effects of PVEc on global uptake measures of [^18^F]FDG- and [^18^F]AV45-PET data and their derived covariance network reconstructions for differentiating between patients and normal older subjects. Network organization patterns were assessed using graph theory in terms of “degree”, “modularity”, and “efficiency”. PVEc evidenced effects on global uptake measures that are specific to either [^18^F]FDG- or [^18^F]AV45-PET, leading to increased statistical differences between the groups. PVEc was further shown to influence the topological characterization of PET-derived covariance brain networks, leading to an optimised characterization of network efficiency and modularisation. Partial-volume effects correction improves the interpretability of PET data in AD and leads to optimised characterization of network properties for organisation or disconnection.

**Electronic supplementary material:**

The online version of this article (10.1007/s11682-019-00247-9) contains supplementary material, which is available to authorized users.

## Introduction

Alzheimer’s disease (AD) is a main neurodegenerative disorder and the most common form of dementia in older persons https://www.alz.org/. Neurodegeneration can be understood as a progressive loss of nerve cells with characteristic histological damage patterns; often underlying an aggregation-prone misfolding of proteins (Jeong 2017; Lane et al. 2018). Several studies have demonstrated that magnetic resonance imaging (MRI)- and positron emission tomography (PET)-derived markers of disease, including atrophy and amyloid load and regional cerebral hypometabolism, are sensitive indicators of disease state and disease stage (Grothe et al. 2017; Jack et al. 2018; Sakr et al. 2019). Therefore as common indicators about neuronal injury, synaptic dysfunction and the degree of neurodegeneration in AD research (Jack et al. 2018; Shokouhi et al. 2013).

It is currently accepted that AD symptoms are due to regional vulnerability to cellular neurodegeneration, amyloid protein accumulation and to disconnection of distant cortical regions (Daianu et al. 2013; Prescott et al. 2014). Recent evidence has highlighted divergent patterns in the spatial evolution of amyloid pathology and neurodegeneration, where atrophy is considered to be mostly driven by tau pathology (Grothe et al. 2016; Iaccarino et al. 2018; Perani 2014) given that local grey matter (GM) atrophy (La Joie et al. 2012; Villain et al. 2010) and tau deposition (Bischof et al. 2016; Chiotis et al. 2018) correlate with FDG-PET evidenced hypometabolism.

In previous studies we and others have shown that partial volume effects (PVE)-correction (PVEc) improves the interpretation of amyloid PET data, by reducing noise measurement in the GM tissue and increasing group discrimination between healthy older people and AD patients (Brendel et al. 2015; Gonzalez-Escamilla et al. 2017; Rullmann et al. 2016; Su et al. 2016). While increased group discrimination after PVEc may not to be the case for ^18^F-fluorodeoxyglucose (FDG)-PET data, in which the magnitude of the group differences results from a combination of both genuine metabolic reductions and negative effects of PVE on the FDG-PET signal, thought to be driven by increased atrophy in the patient group (Meltzer et al. 1996). However, only little is known about how different molecular imaging-derived AD hallmarks, recapitulating disease-related brain abnormalities, relate to network characteristics and how they add to the understanding of reorganization patterns due to disease.

Because PET imaging offers a unique opportunity assess molecular brain processes in vivo, recent efforts have focused on how the study of regional interrelations (i.e. covariance) in the PET signal can complement our current understanding of the accumulation of AD pathology (Arnemann et al. 2018; Carbonell et al. 2014; Huang et al. 2010; Titov et al. 2017). An emerging approach for studying disease-related large-scale topological re-organization is graph theory (Bullmore and Sporns 2009). Studies using graph theory analysis based on molecular imaging have recently shown disrupted networks in AD patients in comparison to cognitively healthy age matched controls (CN) for glucose metabolism and amyloid tracers (Chung et al. 2016; Pereira et al. 2018; Sanabria-Diaz et al. 2013; Seo et al. 2013; Son et al. 2015; Yang et al. 2017; Yao et al. 2010). Nevertheless, the reliability of PET measures greatly depends on the use of quantification methods (Cohen et al. 2013; Villemagne et al. 2012). In this sense, most of these studies have attributed changes in network topology to the disease, without considering possible effects introduced by methodological constraints of the PET images, leading to inconclusive results and some discrepancies in the reported direction of changes in local and global metrics between control and AD groups. Moreover, only little attention has been paid to the possible benefits of PVE correction to quantitatively study PET-based covariance networks (Yang et al. 2017). Since, factors such as the use of different PET tracers for studying the same molecules (e.g., based on Carbon-11 (^11^C) or Fluorine-18 (^18^F) for amyloid imaging), and the degree of brain atrophy showing differential effects over distributed brain regions (Shidahara et al. 2017; Stam 2014) may impact the findings. Knowledge on how PVE affects the regional covariance patterns and its impact on the quantification of topological organization patterns is essential for a better understanding of molecular network alterations in AD.

Therefore, we provide an in-vivo characterization of the condition- specific effects of PVEc for metabolic and amyloid PET imaging quantitation and use graph theory to further investigate the effects of PVEc on PET-derived network topology. Based on the existing differences between the biological processes as measured with amyloid- and FDG-PET tracers, we hypothesize that disease-specific topological network patterns should arise. We assume that: 1) we assume that PVEc affects the data depending on the underlying, investigated biology, 2) a spatial association between amyloid deposition or hypometabolism and neurodegeneration exist and further, that this regional associations spatially correspond with indicators of regional network susceptibility (degree), 3) the distributed pattern of high accumulation, detected by brain amyloid PET, should result in a globally less efficient and less modular network in AD compared to CN; whereas hypometabolism, known to occur in a more focalized set of regions, should result in more structured networks. Here, we expect network differences before and after partial volume effects correction due to confounding disease-related factors on raw data. Altogether, PVEc should enhance group differentiation, thus, improving the interpretability of PET-derived networks. In order to test our hypotheses, we calculated molecular covariance networks for [^18^F]AV45- and [^18^F]FDG-PET data, as the correlation strength between pairs of brain regions, across patients and normal older subjects. The network topological architecture was characterized by the metrics “degree”, “modularity”, “local efficiency” and “global efficiency” (cf. “Materials and Methods/ *Region-based uptake covariance and network metrics*” section).

## Materials and methods

Data used in the preparation of this article were obtained from the Alzheimer’s Disease Neuroimaging Initiative (ADNI) database (adni.loni.usc.edu). The ADNI was launched in 2003 as a public-private partnership, led by Principal Investigator Michael W. Weiner, MD. The primary goal of ADNI has been to test whether serial magnetic resonance imaging (MRI), PET, other biological markers, and clinical and neuropsychological assessment can be combined to measure the progression of mild cognitive impairment (MCI) and AD.

### Subjects and imaging data

Detailed explanation of the ADNI imaging data retrieval is given in (Gonzalez-Escamilla et al. 2017). ^18^F-florbetapir ([^18^F]AV45)-PET, ^18^F-deoxyglucose ([^18^F]FDG)-PET and structural MRI scans were retrieved from already well-characterized CN subjects and AD patients clinically diagnosed with AD dementia, see M. J. Grothe et al. (2018) for details on sample selection. General diagnostic procedures as well as inclusion and exclusion criteria for the selected ADNI cohort have been previously reported (M. J. Grothe et al. 2018). Written informed consent was obtained from all study participants according to the Declaration of Helsinki, and ethical approval for data collection and sharing was given by the institutional review boards of the participating institutions in the ADNI study.

According to validated cut-off threshold for [^18^F]AV45-PET standard uptake value ratios (SUVR) in the ADNI cohort, subjects were divided into positive- (SUVR >1.11) and negative-amyloid (SUVR <1.11) (Landau et al. 2012). To better dissect the effects of amyloid and diagnosis on SUVR and network topology, only AD patients with PET evidence of cerebral amyloidosis were included in the patient sample (*N* = 75), whereas the control sample (*N* = 126) consisted of CN participants with no evidence of cerebral amyloidosis.

### Image processing

MRI data was processed using the SPM8 software (http://www.fil.ion.ucl.ac.uk/spm/) and the intensity-based segmentation algorithm from the VBM8-toolbox (http://dbm.neuro.uni-jena.de/vbm/). In brief, segmentation into GM, white matter (WM) and cerebrospinal fluid (CSF) tissue compartments in subject’s native space was obtained from the intensity distribution of the image and using an adaptive Maximum a Posterior (AMAP) approach (Rajapakse et al. 1997) with partial volume estimation (Tohka et al. 2004), and further refined by applying an iterative hidden Markov random field model (Cuadra et al. 2005) to remove isolated voxels unlikely to be assigned to a determinate tissue type. Intensity values in the resulting maps represent a probability to belong to a pure tissue type (Gaser 2009).

Given that structural brain characteristics change considerably in advanced age and AD, and spatial registration accuracy worsens with deviance from the template characteristics, the tissue maps of each subject were spatially normalized to an aging/AD-specific reference template (M. Grothe et al. 2013) using high-dimensional warping with DARTEL (Ashburner 2007). This template was derived by DARTEL-alignment of 50 healthy old subjects and 50 subjects with very mild, mild and moderate AD retrieved from an open access MRI database (http://www.oasis-brains.org), and is intended to reflect unbiased aging/AD-specific structural characteristics.

**Fig. 1:**
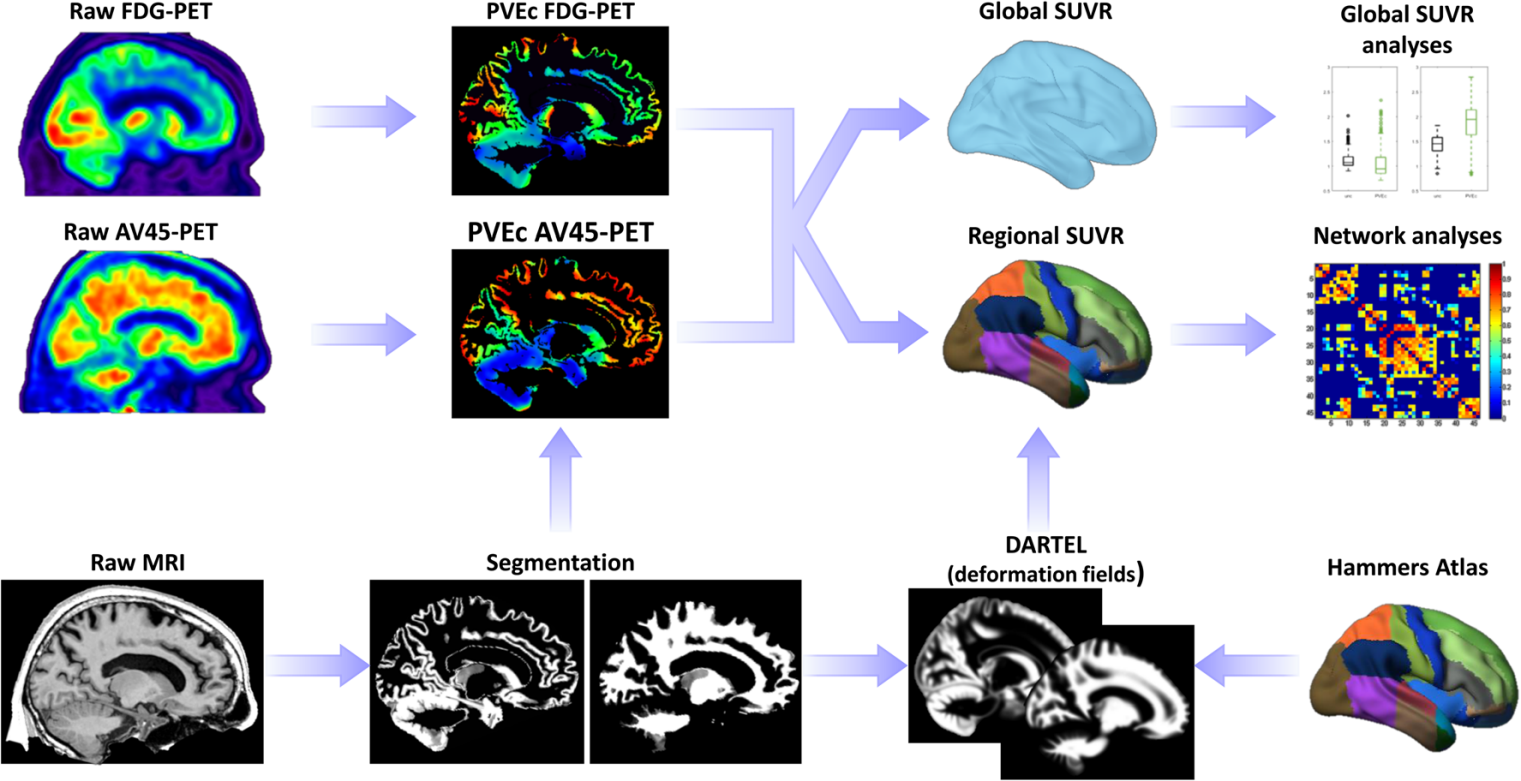
Study pipeline. Firstly, intra-subject registration of the PET images into the space of the subject’s T1-weighted MRI image is effectuated, followed by partial volume effects correction (PVEc) using the PETPVE toolbox for SPM. The spatial normalization parameters based on the DARTEL deformation are calculated on the corresponding MRI data and used to deform the brain parcellation (75 regions from the Hammers atlas) into the individual PET space allowing the computation of average global and regional standardized uptake value ratios (SUVR) used for subsequent SUVR and network analyses

The MRI co-registered [^18^F]FDG- and [^18^F]AV45-PET scans in native space were corrected for PVE using the 3 compartment algorithm (Muller-Gartner et al. 1992) as implemented in the PETPVE-toolbox (Gonzalez-Escamilla et al. 2017). In brief, assuming that the PET signal within WM and CSF compartments is homogeneous, the mean tracer uptake is computed within the respective tissue maps (threshold at 99% tissue probability). This signal is assigned to its respective compartment mask and convolved by the point spread function of the PET scan (8 mm^3^ for ADNI scans). Spill-in effects of WM/CSF signal into the GM are corrected by subtracting the convolved maps of WM/CSF PET activity form the original PET scan. Spill-out effects of GM signal into WM/CSF compartments are corrected by dividing the spill-in corrected PET scan by a convolved version of the GM map. Only regions with a GM probability of at least 50% were retained in the PVE corrected versions of the [^18^F]FDG- and [^18^F]AV45-PET scans. Figure 1 illustrates the image processing workflow.

Whole-brain patterns of AD-typical amyloid deposition and hypometabolism were additionally estimated by computing Z-score maps (z-map) as the mean difference between AD patients and CN scaled by the standard deviation of the CN group at each voxel using pre-processed PET scans.

### PET uptake values

The specific effects of PVE-correction were assessed via global and regional uptake values (see “Supplementary information”) corresponding to the cortical and subcortical brain regions defined in the Hammers Maximum Probability atlas (Hammers et al. 2003), while excluding the ventricles and cerebellum. The resulting 75 regional masks were transformed to native space by inverting the DARTEL flow-fields derived from the processing of the corresponding MRI scans. Before uptake extraction, the native cortical masks were restricted to the individual’s GM map (50% tissue probability threshold). Then, the average uptake across all voxels covered by the masks was computed to obtain a global mean uptake value for each PET tracer.

To minimize inter-subject variability, the regional and global [^18^F]FDG- and [^18^F]AV45-PET uptake means were converted to SUVRs by scaling to the mean uptake value within a mask of the cerebellum derived from the same atlas.

For [^18^F]AV45- and [^18^F]FDG-PET measurement analyses, the effect of PVEc on global brain SUVRs was assessed qualitatively using Bland-Altman plots, i.e. by plotting the difference between the PVEc and non-corrected values against the mean of the two values. The correspondence among the PVE-corrected and non-corrected SUVRs was assessed by the nonparametric Kendall’s W test (Kendall and Smith 1939) and confirmed by Pearson correlation analysis. Inter-subject variability was determined by the coefficient of variation (%COV = standard deviation/mean × 100%).

### Region-based uptake covariance and network metrics

Regional [^18^F]AV45- and [^18^F]FDG-PET SUVRs were used to calculate molecular covariance networks, as the correlation strength (network edges) between pairs of brain regions (network nodes), computed across individuals within each group. The connection between regions is thus given by their shared molecular properties, i.e., level of amyloid deposition or glucose metabolism. For each PET modality/group, this procedure resulted in 75 × 75 covariance matrices.

To quantify between group differences in molecular network organization, first, each covariance matrix was binarized with a minimum density threshold. The minimum density is calculated as the proportion of connections that allow the network of each group to be fully connected, avoiding the evaluation of fragmented networks. This value (0.6 in our study) is used as starting point for computing network metrics at further densities (Hosseini et al. 2012), and ensures that group differences are not confounded by differing numbers of nodes and edges as for an absolute threshold at a single density. Subsequent network metrics were computed across 20 densities in steps of 5% using the Brain Connectivity Toolbox (BCT, (Rubinov and Sporns 2010)).

Based on recent studies using PET data to compare HC and AD individuals (Chung et al. 2016; Duan et al. 2017; Pereira et al. 2018; Sanabria-Diaz et al. 2013; Yang et al. 2017), the following metrics were computed to characterize the network topological architecture:

The *degree* of a region, the most fundamental metric commonly known as its centrality, is equal to the number of edges connecting that region to the rest of the network (Rubinov and Sporns 2010), and allows to characterize the node importance in the network.

*Modularity*, considered the main measure of network segregation, reflects the degree to which the network may be subdivided into clearly delineated and non-overlapping groups of nodes, with a maximally possible number of within group links, and sparsely connected to the rest of the network (Sporns and Betzel 2016).

Another related metric is the *local efficiency*, which is the ratio of the number of connections between each node’s neighbours to the total number of possible connections between them. This metric is predominantly associated with short-range connections among nearby regions that mediate modularized pathology accumulation or tolerance to network damage (Vito Latora and Marchiori 2003; Vragovic et al. 2005).

Given that the covariance paths represent sequences of statistical associations and may not correspond to anatomical connections, the characteristic path length metric is not straightforward to interpret (Rubinov and Sporns 2010). A more meaningful metric is the *global efficiency* (V. Latora and Marchiori 2001), reflecting how efficiently the information can be exchanged over the network, is computed as the average of the efficiencies over all network nodes. Unlike the path length, the global efficiency can be meaningfully measured on both fully connected and disconnected networks, where lower values indicate weaker connections between modules, hence a less integrated network.

Formal mathematical definitions of the metrics can be found in the supplementary information according to the work of Rubinov and Sporns (2010).

### Statistical analyses

Demographics and cognitive scores were compared between diagnostic groups using two-sample t-tests (for continuous variables) and chi-square tests (for categorical variables). Differences in global SUVRs were assessed using analysis of variance (ANOVA) with factors group (CN and AD) and method (non-corrected and PVEc) applying the Bonferroni method (*p* < 0.05) at the post hoc analyses. In addition, effect sizes (Cohen’s d’) were calculated for group differences (CN vs AD) in both non-corrected and PVEc data.

To create a reference for the local associations between the patterns of amyloid pathology or hypometabolism with neurodegeneration, association maps were created by computing the partial correlation across AD patients between each PET z-map (cf. “Materials and Methods/ *Image processing*” section) and the MRI-derived GM-atrophy z-map at each voxel, while correcting for the effects of age, gender and MMSE. In doing so, the change of MRI-derived GM volume with respect to controls, is considered as a consequence of neurodegeneration; this term is utilized in this manner throughout this manuscript.

For each PET modality we assessed the potential for degree, modularity, local and global efficiency to differentiate between CN and AD groups. Group differences in network characteristics were assessed by taking the grou*p* values across all network densities into two-sample t-tests. The area under the curve (AUC) was additionally computed across network density thresholds (cf. supplementary methods) to provide a summary *p* value of the between-groups difference (pAUC). An advantage of this approach is that taking into account the whole set of thresholds at the same time, the problem of multiple testing per threshold is avoided.

## Results

Demographic and clinical characteristics and group differences of the included ADNI participants are summarized in Table 1. The full characteristics of this population have been described previously (M. J. Grothe et al. 2018). APOE genotype was not available for eight participants (3 CN and 5 AD).

**Table 1:** Demographic characteristics of the study cohort

	CN	AD	statistic	p value
n	126	75		
Age in years	72.7 ± 6.4	75.0 ± 8.5	2.05	**0.043**
Sex (F/M)	65/61	35/40	0.06	0.8
Education, years	16.8 ± 2.5	15.6 ± 2.8	3.2	**0.002**
APOE4 (%)	22	79	58.53	**<0.001**
CDR (0/0.5/1/2/3)	126/0/0/0/0	0/32/42/1/0	N/A	N/A
CDR-SOB	N/A	4.5 ± 1.5	N/A	N/A
MMSE	29.1 ± 1.2	22.9 ± 2.1	18.2	**<0.001**

Average values are reported as mean ± SD

Demographic variables and cognitive scores were compared two-sample t-tests

Categorical variables were analysed using chi-square tests

CN = cognitively normal controls; AD = Alzheimer’s disease dementia patients; CDR-SOB = Clinical Dementia Rating – Sum of Boxes; F = female; M = male; MMSE = Mini-Mental State Examination; n = sample size; N/A = not applicable

Comparisons between diagnostic groups were carried out using two-sample t-tests for continuous variables, and chi-square tests for categorical variables

### Effects of partial volume correction on PET data

**Fig. 2:**
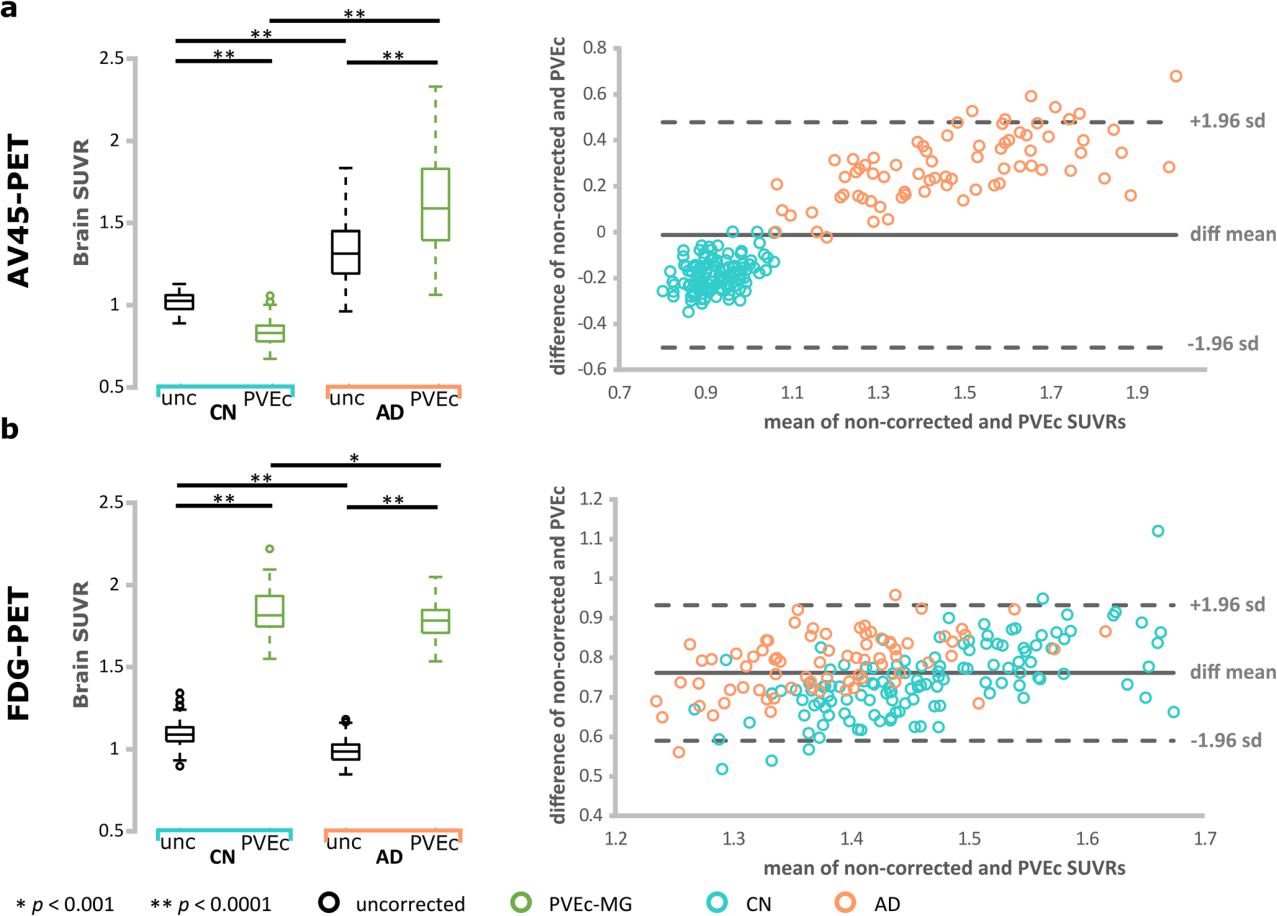
Global standard uptake value ratios (SUVR) analysis results for (a) [^18^F]AV45-PET and (b) [^18^F]FDG-PET. Left side: Box-plots depicting the distribution of the data before and after partial volume effects correction (PVEc) and further indicating the increased variability outside upper and lower quartiles after PVEc. On each boxplot, the central mark indicates the median, and the bottom and top edges of the box the upper (75th) and lower (25th) percentiles, the whiskers extend to the most extreme points of the data distribution that are not considered as outliers, while outliers are plotted beyond with a circle. Right side: Bland-Altman plots showing the agreement between non-corrected (unc/uncorrected) and PVE-corrected PET images. * indicates significant differences in the ANOVA model after correcting for multiple comparisons in the post hoc analyses (Bonferroni, *p* < 0.05). The light blue colour indicates healthy control (CN) subjects; orange colour indicates Alzheimer’s disease dementia (AD) subjects

For [^18^F]AV45-PET data PVEc yielded lower SUVR values for the CN group (−18.4%, *p* < 0.0001) and higher SUVR values for the AD group (+21.2%, *p* < 0.0001) with respect to the non-corrected data (Fig. 2, boxplots), which is in line with previous studies (Brendel et al. 2015; Gonzalez-Escamilla et al. 2017; Su et al. 2016; Su et al. 2015). The inter-subject variability of the amyloid data in terms of %COV was increased after PVE-correction (CN: from 5.3 to 8.9 %COV; AD: from 14.1 to 17.3 %COV). Group differences (CN vs. AD) using non-corrected data (T = 11.4, *p* < 0.0001, Cohen’s d’ = 2.26) were also increased after PVEc (T = 16.2, *p* < 0.0001, Cohen’s d’ = 3.85). For [^18^F]FDG-PET PVEc increased global SUVR values for both the CN (+68.3%, *p* < 0.0001) and AD (79.9%, *p* < 0.0001) group. Using this tracer, the inter-subject variability was only slightly increased after PVE-correction in CN (from 6.5 to 6.7 %COV) but decreased in AD (from 6.8 to 5.8 %COV). Group differences (CN vs. AD) using non-corrected data (T = 10.2, *p* < 0.0001, Cohen’s d’ = 1.51) were also increased after PVEc (T = 3.85, *p* = 0.00016, Cohen’s d’ = 0.55). The same trend was shown during the regional analyses (see supplementary information). Confirming previous suggestions that PVEc exerts differential effects depending on the investigated underlying processes (Gonzalez-Escamilla et al. 2017).

The Bland-Altman-Plots for [^18^F]AV45- and [^18^F]FDG-PET SUVR values (Fig. 2, right side) also depicted tracer-specific effects for PET data. For [^18^F]AV45-PET the mean and standard deviation of differences were − 0.01 and 0.25 and the 95% confidence interval was given by [−0.5;0.48], evidencing a good relation between the difference in the measurements and their mean value. The concordance and correlation analyses revealed a strong correspondence between the corrected and non-corrected values as indicated by W = 0.92 (*p* = 4.8e-12) and rho = 0.926 (*p* = 7.1e-86).

For the [^18^F]FDG-PET data, the mean and standard deviation of differences were 0.76 and 0.09 and the 95% confidence interval was given by [−0.59;0.93]. The Bland-Altman-Plot depicted a slight relation between the difference in the estimates and their mean values; differences between methods are throughout positive. The concordance and correlation analyses also showed good correspondence between the corrected and non-corrected data as given by W = 0.84 (*p* = 7.8e-9) and r = 0.685 (*p* = 3.3e-29).

### Spatial association between amyloid deposition or hypometabolism and neurodegeneration and impact of PVEc on network degree

**Fig. 3:**
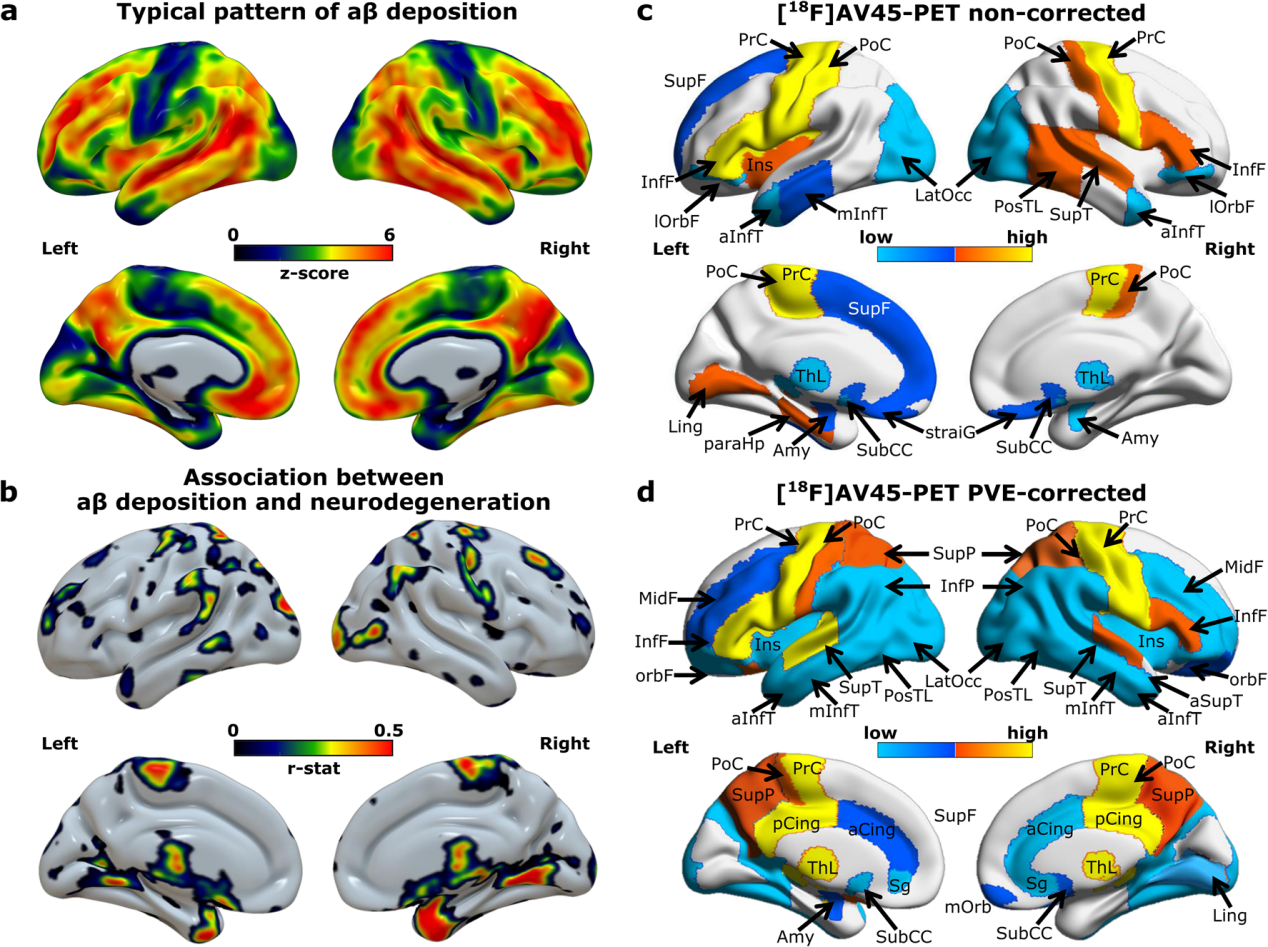
Local network characteristics in the [^18^F]AV45-PET amyloid network. (a) Z-maps depicting the typical brain patterns of amyloid deposition in Alzheimer’s disease. (b) Association maps showing the specific distribution of the associations between amyloid deposition and neurodegeneration. The maps express the partial correlation (r-score) between each [^18^F]AV45-PET (z-score) and the MRI-derived GM-atrophy (z-score) at each voxel after FDR correction, while accounting for the effects of age, gender and MMSE. (c, d) Regions showing significant network susceptibility (degree centrality) before and after partial volume effects correction (PVEc). All presented regions survived after correction for multiple comparisons with FDR (*p* < 0.05). The cold colour scale indicates the regions with low suceptibility, while the hot colour scale indicates high suceptibility. SupF, superior frontal; InfF, inferior frontal; PosTL, posterior temporal lobe; PrCG, precentral; PoC, postcentral; antSupT, anterior superior temporal; aInfT, anterior inferior temporal; mInfT, middle and inferior temporal; lOrbF, lateral orbitofrontal; mOrb; medial orbital; OrbF, orbitofrontal cortex; paraHp, parahippocampus; straiG, Straight gyrus; pCing, posterior cingulate; aCing, anterior cingulate; Ling, Lingual; ThL, Thalamus; Ins, Insula; SubCC, subcallosal area; Amy, amygdala; Sg, pre−/subgenual anterior cingulate; InfP, inferior parietal; SupP, superior Parietal

**Fig. 4:**
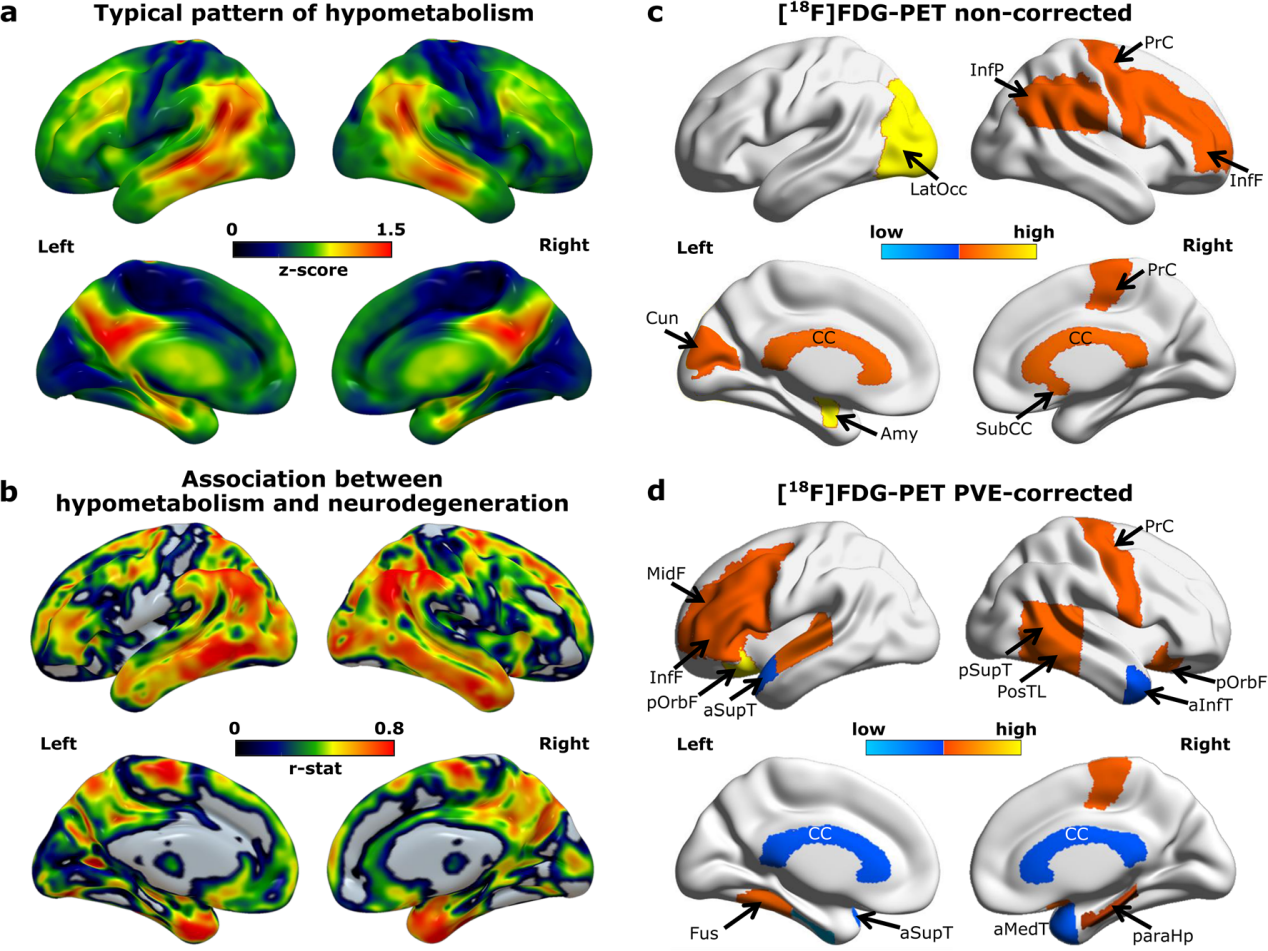
Local network characteristics in the [^18^F]FDG-PET metabolic network. (a) Z-maps depicting the typical brain patterns of glucose hypometabolism in Alzheimer’s disease. (b) Association maps showing the specific distribution of the associations between hypometabolism and neurodegeneration. The maps express the partial correlation (r-score) between each [^18^F]FDG-PET (z-score) and the MRI-derived GM-atrophy (z-score) at each voxel after FDR correction, while accounting for the effects of age, gender and MMSE. (c, d) Regions showing significant network suceptibility (degree centrality) before and after partial volume effects correction (PVEc). The cold colour scale indicates the regions with low suceptibility in the network topology, while the hot colour scale indicates high suceptibility. PosTL, posterior temporal lobe; PrCG, precentral gyrus; antSupT, anterior superior temporal; aInfT, anterior inferior temporal; aMedT, anterior medial temporal; pOrbF, posterior orbitofrontal; InfP, inferior parietal; paraHp, parahippocampus; Amy, amygdala; Fus, fusiform; SubCC, subcallosal area; LatOcc, lateral occipital; CC, corpus callosum

To determine the ability of network analysis in depicting local changes related to AD, first, the degree of each brain region was compared between the groups, evidencing regional differences in the network organization for both PET modalities. For [^18^F]AV45-PET most of the regions showing high amyloid deposition (Fig. 3a) show a different pattern than the regions associated with neurodegeneration (Fig. 3b), and high overlap with the regions showing lower degree centrality (i.e. lower network vulnerability) after PVEc (Fig. 3d) but not before (Fig. 3c). The non-corrected amyloid data showed less structures (31 regions) with significant connectivity differences than after PVE-correction (52 regions). For [^18^F]FDG-PET again the regions showing the known AD-pattern of hypometabolism (Fig. 4a) overlapped with regions associated with neurodegeneration (Fig. 4b) and, at some extent, with those showing high network vulnerability (Fig. 4c, d). Similarly as for amyloid data the non-corrected network showed connection differences in a lower number of regions (8 regions) than after PVEc (16 regions).

**Fig. 5:**
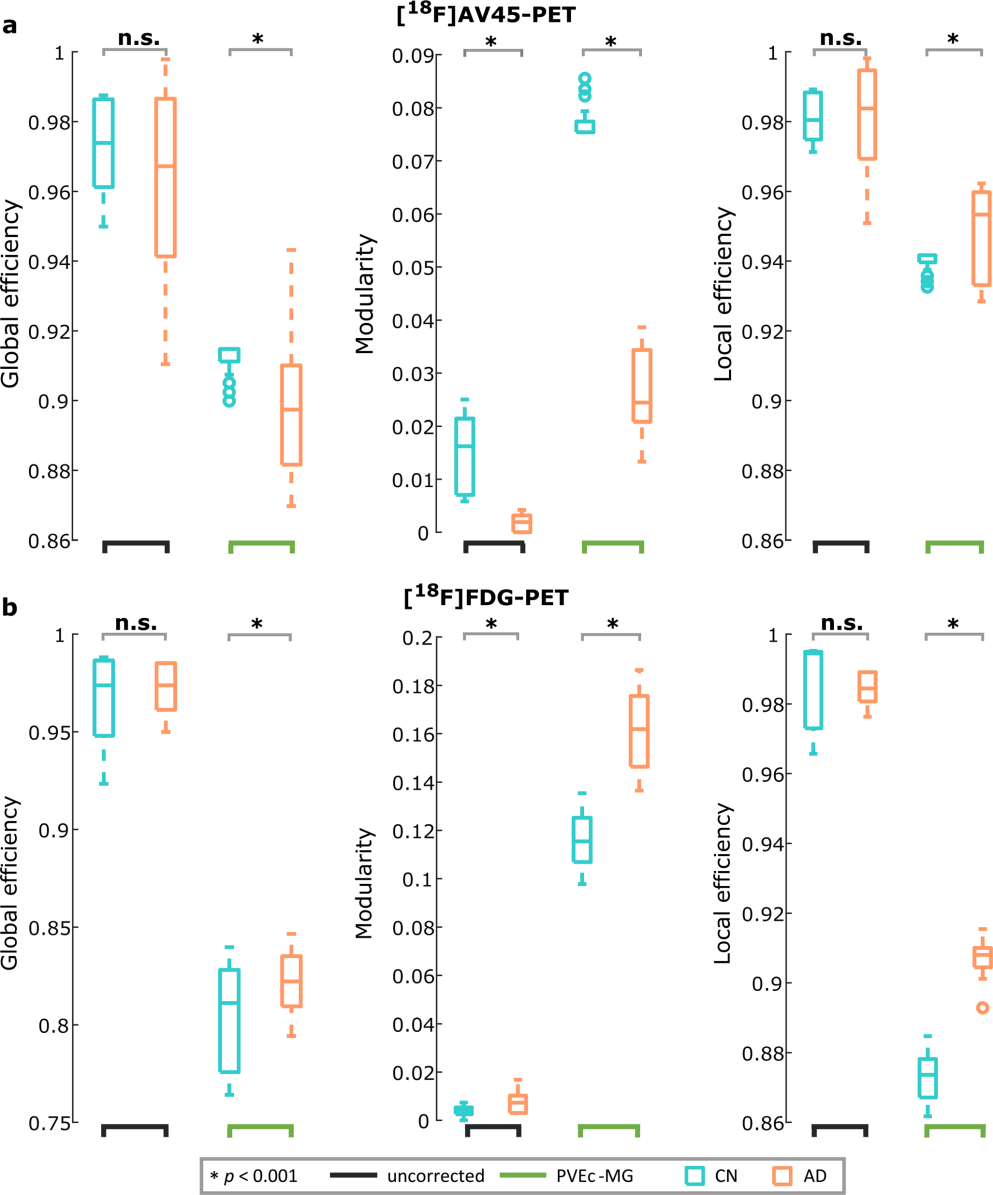
Whole brain network topology differences between Alzheimer and control groups. Box-plots showing the comparison of network measures (across densities) between cognitively normal elders (CN) and Alzheimer’s disease dementia (AD) patients for (a) [^18^F]AV45-PET and (b) [^18^F]FDG-PET data before and after partial volume effects correction (PVEc). On each boxplot, the central mark indicates the median, and the bottom and top edges of the box the upper (75th) and lower (25th) percentiles, the whiskers extend to the most extreme points of the data distribution that are not considered as outliers, while outliers are plotted beyond with a circle. The light blue colour indicates CN; orange colour indicates AD; the black lower line indicates the non-corrected (uncorrected) PET data; and green lower line indicates the PVEc values

For the [^18^F]AV45-PET network (Fig. 5a) the group comparison based on non-corrected data showed decreases in modularity (*p* = 3.1e-8, t_199_ = 6.67; pAUC <0.001) in AD subjects, but no significant differences in global efficiency (*p* > 0.05, t_199_ = 1.53, pAUC = 0.07) or local efficiency (*p* > 0.05, t_199_ = 0.02, pAUC =0.5) between groups. In AD patients PVEc evidenced decreased modularity (*p* = 9.3e-20, t_199_ = 16.8, pAUC <0.001), and decreased global efficiency (*p* = 0.001, t_199_ = 3.3, pAUC <0.001), and local efficiency (*p* = 0.02, t_199_ = 2.13, pAUC = 0.02).

### Impact of PVEc on network modularity, local and global efficiency

For the [^18^F]FDG-PET network (Fig. 5b), the non-corrected data showed increases in modularity (*p* = 0.0009, t_199_ = 3.34, pAUC = 0.05) in the AD group compared to the CN group and no difference in global efficiency (*p* > 0.05, t_199_ = 1.32, pAUC = 0.14) or local efficiency (*p* > 0.05, t_199_ = 0.12, pAUC = 0.43). The PVEc data showed significantly increased modularity (*p* = 5.4e-12, t_199_ = 9.5, pAUC <0.001), and increased global efficiency (*p* = 0.008, t_199_ = 2.51, pAUC = 0.05), and local efficiency (*p* = 2.6e-19, t_199_ = 16.3, pAUC <0.001) in AD compared to CN.

## Discussion

In this study we aimed at characterising network organization in AD. We first evaluated the specific effects of PVEc for detecting group differences for two of the most used PET tracers in AD research, [^18^F]AV45 and [^18^F]FDG, indicators of amyloid pathology and hypometabolism, respectively. Evidencing that regions showing increased network degree in comparison to other regions corresponded to the typical patterns of amyloid deposition and hypometabolism, respectively, and in turn with regions correlated with neurodegeneration. We then studied the capability of PET-derived covariance networks to differentiate between CN and AD subjects. we also examined the effects of PVEc on the topological organization of the networks.

### Effects of partial volume correction on PET data

In our study, the signal changes after PVEc were accompanied by an increase of inter-subject variability, which was more pronounced in [^18^F]AV45-PET data probably due to the high difference in tracer uptake between tissues, specifically in the WM, compared to [^18^F]FDG-PET data. The increase of variability after PVEc has been previously reported for the MG PVEc method (Thomas et al. 2011), and seems to be also common to different PVEc methods (Harri et al. 2007). Indeed, an overall increase in [^18^F]FDG SUVRs after PVEc has been previously shown as a common result for [^18^F]FDG-PET data (Meltzer et al. 1996). For [^18^F]AV45-PET this increase is only seen in AD subjects, whereas reduced SUVR is commonly detected in CN subjects (Brendel et al. 2015; Gonzalez-Escamilla et al. 2017). Therefore, it can be then argued that the observed increase in between group global SUVR differences after PVEc is an indicator of increased representation accuracy of the data and not a correction-induced error, i.e. increased image noise (Thomas et al. 2011), see (Gonzalez-Escamilla et al. 2017) for a detailed explanation on these effects.

It has been recently postulated that the spill-out effects on PET imaging are influenced not only by the size of the measured GM region (Hoffman et al. 1979), thus increased by brain atrophy, but also influenced by the effect of spill-out and spill-in relationship of the specific tracer binding between GM and WM tissue (Gonzalez-Escamilla et al. 2017) and subject condition (health/disease) (Shidahara et al. 2017). Hence, the effects of PVEc may be specific to the underlying biological process. This hypothesis is supported by our results, in which for example, after PVEc the [^18^F]AV45-PET signal presented reductions in subjects with low tracer binding (i.e., CN subjects) and increases in subjects with high amyloid load, i.e. AD subjects. PVEc in amyloid sensitive PET data has been shown to improve its utility when used to compare healthy and AD groups (Gonzalez-Escamilla et al. 2017; Yang et al. 2017), and currently corroborated by the increased effect sizes. On the contrary, in the case of [^18^F]FDG-PET PVE-correction does not seem to improve the ability for group differentiation (Ibanez et al. 1998; Meltzer et al. 1996; Samuraki et al. 2007), and also shown here by slightly reduced effect sizes, which can be due to the general increased signal in all subjects after PVEc, as shown here. A critical factor between the two radiotracers used in the current study is the degree of unspecific white matter binding, which can also contribute to some of the differences reported in this study. Hence, the role of tracer-specific off-target binding should be studied more in detail in further studies.

The differential effects of PVEc on different PET tracers can be further related to their capability to depict regional vulnerability to cellular neurodegeneration or amyloid protein accumulation. Then, for tracers such as [^18^F]FDG-PET which are highly related to the underlying brain anatomy (Horwitz et al. 1984) and correlate with local GM atrophy and Tau deposition, by contrast to amyloid tracers (Bischof et al. 2016; La Joie et al. 2012; Villain et al. 2010), it can be expected that the PVEc causes the global PET signal to be more homogeneous across subjects and groups. This is demonstrated by the low increase in inter-individual variability in both groups and no improvement of SUVRs to differentiate between groups after PVEc. In contrast, for [^18^F]AV45-PET low to middle ranged SUVR values (corresponding to CN) were reduced, whereas high SUVR values (present in AD) were increased after PVEc, facilitating group differentiation. Notably, both PET tracers showed good concordance after PVEc with their respective non-corrected data.

### Spatial association between amyloid deposition or hypometabolism and neurodegeneration and impact of PVEc on network degree

Accurate estimation of Aβ burden is critical for a better understanding of underlying disease mechanisms, given its relationship with cortical thinning (Dickerson et al. 2008), hippocampal atrophy (Andrews et al. 2013; Chetelat et al. 2012), disruption of functional and structural connectivity (Drzezga et al. 2011; Horwitz et al. 1984; Mormino et al. 2011; Palmqvist et al. 2017; Racine et al. 2014; Sheline et al. 2010), association with metabolic connectivity patterns (Carbonell et al. 2014), and its inversed u-shaped relationship with hypometabolism across disease stages (Kadir et al. 2012; Mosconi and McHugh 2011). Previous studies using SUVR-based univariate analyses in AD have shown characteristic patterns of hypometabolism and increased cerebral amyloid at sets of distributed brain regions, which in turn differ between tracers (M. J. Grothe et al. 2016; La Joie et al. 2012; Perani 2014). One of the purposes of applying PVEc on PET data is to enhance the sensitivity of detecting regional changes by attenuating the bias induced by the concomitantly progressing cortical atrophy, which leads to underestimation of the SUVR in non-corrected PET data (Brendel et al. 2015; Erlandsson et al. 2012; Rullmann et al. 2016; Su et al. 2015). The definite improvement of PVEc for both PET tracers, was evidenced by the increased regional correspondence between AD pathology, high network degree centrality (depicting increased network susceptibility or vulnerability) and the correlation with neurodegeneration. Furthermore, the regional analyses (see supplementary information) demonstrated that, even when no between region spill-over corrections are performed by PVEc, different regions showed different trends. Altogether, the our results argue against possible systematic bias introduced by PVEc on both PET data. Despite the apparent lack of quantitative improvement for global [^18^F]FDG-PET SUVR values by PVEc, the relationship with local atrophy suggests the regional quantitation to be improved, and PVEc may be important for analysis methods requiring regional quantitation. Therefore, reports based on non-corrected data may also bias by the amount of structural atrophy of the selected participants, which limits the interpretability of the results if the local associations between tracer binding and neurodegeneration are not considered.

### Impact of PVEc on network modularity, local and global efficiency

In accordance with previous studies, our molecular networks showed prominent topographical differences between CN and AD patients, indicating a change in the balance of integration and segregation related to the disease condition. Notably, PVEc over FDG- and AV45-PET data resulted generally in no changes in the directionality of the between groups differences for network measures, but in increased sensitivity to detect the group differences. For the [^18^F]AV45-PET the finding of reduced global efficiency in the AD group, in the non-corrected but also after PVEc, is consistent with recent reports of Yang and co-workers (Yang et al. 2017), but comparison of the modular organization with previous studies (Pereira et al. 2018; Yang et al. 2017) is not possible given the lack of this metric in those studies. In the case of [^18^F]FDG-PET there is a lack of consensus, where decreases, increases and no changes in the local measures of network topology (local efficiency) have been reported between CN and AD groups (Chung et al. 2016; Sanabria-Diaz et al. 2013; Seo et al. 2013).

Of note, no previous studies have reported on the modular structure of the molecular networks, a hallmark measure when studying the organization of complex networks (Sporns and Betzel 2016). Those studies have rather focused on the small-world properties of the network. However, covariance networks are based on the idea that if the molecular properties of two regions are statistically associated with each other then they are connected. Such associations do not necessarily imply the existence of anatomical connections between network nodes (regions) and the paths may transverse regions with low correlation weights (Fornito et al. 2016), making the path lengths of the network difficult to interpret (Rubinov and Sporns 2010). Care must therefore be taken when interpreting small-world and related measures.

Related to the fact that hypometabolism and amyloid deposition reflect different aspects and patterns of regional vulnerability to AD pathology (M. J. Grothe et al. 2016; La Joie et al. 2012; Perani 2014), the direction of group differences for the measures of network organization in both [^18^F]FDG- and [^18^F]AV45-PET data was opposed.

In contrast to previous studies we limited our AD sample to include only amyloid-positive patients, hence the strong alterations in network structure can be most likely attributed to the effects of amyloid accumulation and hypo-metabolism. In this respect, the opposite directions of groups differences, in both [^18^F]AV45-PET and [^18^F]FDG-PET network reconstructions, is not surprising. Therefore, decreases in modularity in the [^18^F]AV45-PET, can be though to be related to increased amyloid accumulation in key regions for the communication between different modules (namely network hubs), such as the posterior and anterior cingulate and frontal cortices. Then, disruption of network efficiency can be directly explained by propagated amyloid pathology across the brain region of AD subjects. On the other hand, the increases in modularity for [^18^F]FDG-PET, accentuated after PVEc, match commonly reported findings from structural and functional MRI network studies into the neurodegenerative side of AD(Lopez-Sanz et al. 2017; Pereira et al. 2016). The results are in line with recent studies highlighting the relevance of studying networks derived from [^18^F]FDG-PET in AD (Veronese et al. 2019). This can be directly associated with decreased neural integrity/activity leading to a more segregated topology.

### Limitations

Noteworthy is that the direction of the group differences in modularity and efficiency measures was not inverted after PVEc in either amyloid or metabolic tracers. This further suggests that the network reconstructions from molecular imaging are sensitive to AD pathology and are robust to intrinsic methodological limitations of PET data, which in turn transfer validity to previous reports.

On the regional analyses, PVEc evidences more interpretable results. As an example, the superior parietal/precuneus and posterior cingulate cortices, which before PVEc showed no group differences in degree, turned to present a reduced degree after PVEc relative to the CN group, implying a decrease in the connectivity of these regions in AD patients. These regions are known to be core regions for brain structural and functional networks (van den Heuvel and Sporns 2013), and to be strongly implicated in AD aetiology, being among the main target areas for neurodegeneration and amyloid deposition. The case for the [^18^F]FDG-PET was also similar where the connectivity changes appeared in the medial temporal lobe after PVEc, where hypometabolism in medial temporal areas has been recently suggested as a specific marker for cognitive changes at the earliest stages of the AD continuum due to amyloid pathology (Vannini et al. 2017). These results indicate that the known loss of connectivity previously reported in AD patients using fMRI and structural imaging is based on the underlying molecular properties of the network regions.

## Conclusion

The effects of partial volume effects correction are specific to the underlying biological processes as measured with [^18^F]AV45- and [^18^F]FDG-PET. Increased uptake values for CN and decreased uptake for AD were shown for [^18^F]AV45-PET, whereas for [^18^F]FDG-PET uptake increased for both AD and CN groups. These PVEc effects in turn led to better group differentiation. For the network analyses, PVEc based data analysis indicated that the disruption of network efficiency and modular organization could be directly explained by propagated amyloid pathology and neurodegeneration involving specific brain areas. Therefore, PVEc is of vast importance for PET imaging, especially for characterization of the brain networks using new PET tracers, which in turn opens up new opportunities to study disease trajectories.

## Electronic supplementary material

ESM 1 (DOCX 789 kb)
