# Supplementary material for: Metabolic and amyloid PET network reorganization in Alzheimer’s disease: differential patterns and partial volume effects
Source: Brain Imaging Behav. 2020 Mar 3;15(1):190–204. doi: 10.1007/s11682-019-00247-9 (PMC7835313; doi:10.1007/s11682-019-00247-9)
Supplement: Supplementary file 1 — (DOCX 789 kb) [file 11682_2019_247_MOESM1_ESM.docx]

**Supplementary information**

**Metabolic and amyloid PET network reorganization in Alzheimer’s disease: differential patterns and partial volume effects**

Authors: Gabriel Gonzalez-Escamilla*, Isabelle Miederer, Michel J. Grothe, Mathias Schreckenberger, Muthuraman Muthuraman, Sergiu Groppa.

*Corresponding author: Gabriel Gonzalez-Escamilla, PhD.

Movement Disorders and Neurostimulation, Department of Neurology, Focus Program Translational Neuroscience (FTN), Rhine-Main-Neuronetwork (rmn2), University medical center of the Johannes-Gutenberg-University.

Langenbeckstr. 1. 55131 Mainz, Germany

Tel: +49 6131 17 8080

Email: ggonzale@uni-mainz.de

ORCID: 0000-0002-7209-1736

**Supplementary methods**

Network metric computation according to [Rubinov and Sporns (2010)](#_ENREF_8):

*N* is the set of nodes in the network,
*n* is he number of nodes,

**Degree** of a node *i*:

$$k_{i}=\sum_{j\in N} a_{ij}$$

**Local efficiency** of the network according to [Latora and Marchiori (2001)](#_ENREF_5):

$$E_{\text{loc}}=\frac{1}{n}\sum_{i\in N} E_{\text{loc},i}=\frac{1}{n}\sum_{i\in N} \frac{\sum_{j,h\in N,j\neq i} a_{ij}a_{ih}\left[ d_{jh}\left( N_{i} \right) \right]^{-1}}{k_{i}\left( k_{i}-1 \right)}$$

where E_loc,i_ describes the local efficiency of node *i*,. d_jh_(N_i_) denotes the shortest path length between nodes *j* and *h* containing neighbours of *i*., whereby the shortest path length equals the minimum number of edges that need to be crossed to go from one node to the other.

**Global efficiency** of the network according to [Latora and Marchiori (2001)](#_ENREF_5):

$$E=\frac{1}{n}\sum_{i\in N} E_{i}=\frac{1}{n}\sum_{i\in N} \frac{\sum_{j\in N,j\neq i} d_{ij}^{-1}}{n-1}$$

where E_i_ is the efficiency of node *i*, and it represents the average inverse of the shortest path length between all pairs of nodes in the network.

**Modularity** of the network according to [Newman (2006)](#_ENREF_6):

$$Q=\sum_{u\in M} \left[ e_{uu}-\left( \sum_{\in M} e_{u} \right)^{2} \right]$$

where *M* denotes the set of non-overlapping modules, e_uv_ is the proportion of all connections in module *u* and module *v*.

Furthermore, we calculated the area under the curve (AUC) for each network metric, which provides an integrated metric for topological characterization of brain networks independent of any threshold selection. The integrated AUC metric has been used in previous brain network studies and is sensitive at detecting topological alterations of brain disorders ([Cao et al., 2013](#_ENREF_1); [Drakesmith et al., 2015](#_ENREF_2); [He et al., 2009](#_ENREF_3); [Hosseini, Hoeft, & Kesler, 2012](#_ENREF_4); [Rubinov et al., 2009](#_ENREF_7); [Zhang et al., 2011](#_ENREF_9)). For computing the AUC the group differences at each density are summarized resulting in a curve of significances across computed densities (as depicted in the supplementary Figure 2). Then, the area under this curve is computed. This procedure is repeated for all random network of each group creating a random distribution of permuted AUCs. Finally, the actual between-group AUC difference across all densities was compared with its percentile position in the random AUC yielding the significance level. More specifically the AUC was calculated for each Network metric (*X*) as:

$${AUC}_{X}=\sum_{k=1}^{n-1} \left[ X{(D}_{k}) +{X(D}_{k+1}) \right] \times\Delta D/2$$

where *ΔD* is the density interval [*k* to *n*] and *D_k_* is the minimum density.

Effect sizes of the group differences in regional SUVRs were assessed using the standardized Cohen's d, determined by calculating the mean difference between your two groups, and then dividing the result by the pooled standard deviation:

$$d'=\frac{\bar{\chi}_{GP1}-\bar{\chi}_{GP2}}{\sqrt{\frac{{\mathrm{SD}_{GP1}}^{2}+{\mathrm{SD}_{GP2}}^{2}}{2}}}$$

where *X̅* is the mean SUVR of group 1 (*GP1*) and group 2 (*GP2*), respectively. SD*_GP1_* and SD*_GP2_* are their corresponding standard deviations.

**Supplementary results**

Regional comparisons. The regions were selected a posteriori according to their involvement in AD pathology and the results of the network analyses.

Supplementary Tables 1 and 3 show the comparisons between non-corrected and PVEc for [^18^F]AV45-PET and [^18^F]FDG-PET data, respectively. Supplementary tables 2 and 4 show the comparisons between healthy controls and AD patients within non-corrected and PVEc data. , Supplementary Figure 1 depicts the differences between correction methods and Groups for [^18^F]AV45-PET (Supplementary Figure 1 A) and [^18^F]FDG-PET data (Supplementary Figure 1 B).

Here, the same trends are shown as for global SUVRs at the regional level. Of notice, for [^18^F]AV45-PET the hippocampus and thalamus show decreased instead of increased values after PVEc in AD patients, whereas the effect sizes for distinguishing between HC and AD also increased after PVEc. The same effect on the group discriminability is observed for the global SUVRs and in regions such as the posterior cingulate, superior parietal (including the precuneus) and parahippocampal cortex although in these cases a SUVR increase is observed in AD. For [^18^F]AV45-PET all regions showed very large effect sizes after PVEc, which is not the case when using the non-corrected data. For [^18^F]FDG-PET data, although reduced effect sizes are observed for the comparison between HC and AD after PVEc, the effect sizes remain to be large after PVEc, specifically for the posterior cingulate, superior parietal (including the precuneus) and parahippocampal cortex. The hippocampus remain here to show only medium effect sizes.

Supplementary Figure 2 depict the individual differences between non-corrected and PVEc [^18^F]AV45-PET (Supplementary Figure 2 A) and [^18^F]FDG-PET data (Supplementary Figure 2 B) in each group (CN and AD). Supplementary Figure 3 depict [^18^F]AV45-PET (Supplementary Figure 3 A) and [^18^F]FDG-PET data (Supplementary Figure 3 B) group differences (CN Vs. AD) for non-corrected and PVEc methods.

Altogether, these results confirm our hypothesis on the differential and favourable effects of PVEc at the regional level for both [^18^F]AV45-PET and [^18^F]FDG-PET data.

**Supplementary Table 1**: Comparison of regional SUVRs between non-corrected and PVEc [^18^F]AV45-PET data.

| **Region** | **% SUVR change** | | **p-value** | | **%COV** | |
| --- | --- | --- | --- | --- | --- | --- |
|  | CN | AD | CN | AD | CN | AD |
| posterior cingulate | -15.8 | 19.4 | < 0.0001 | < 0.0001 | 7.4🡪13.1 | 16.0🡪18.4 |
| superior parietal (including the precuneus) | -14.1 | 40.0 | < 0.0001 | < 0.0001 | 7.4🡪15.3 | 15.8🡪21.0 |
| orbitofrontal cortex | 0.3 | 49.4 | = 0.757493 | < 0.0001 | 7.0🡪15.3 | 15.0🡪19.1 |
| parahippocampal cortex | -8.7 | 9.6 | < 0.0001 | < 0.0001 | 7.2🡪12.6 | 14.3🡪15.5 |
| hippocampus | -18.1 | -7.0 | < 0.0001 | < 0.0001 | 6.9🡪8.8 | 15.2🡪14.7 |
| thalamus | -48.2 | -30.2 | < 0.0001 | < 0.0001 | 10.6🡪35.8 | 16.2🡪29.5 |

CN: controls, AD: Alzheimer’s disease patients

**Supplementary Table 2**: Comparison of [^18^F]AV45-SUVR between controls and Alzheimer’s disease patients.

| **Region** | **non-corrected** | | | **PVEc** | | |
| --- | --- | --- | --- | --- | --- | --- |
|  | p | T | Cohen’s d’ | p | T | Cohen’s d’ |
| posterior cingulate | < 0.0001 | -16.3 | 2.61 | < 0.0001 | -23.3 | 3.73 |
| superior parietal (including the precuneus) | < 0.0001 | -17.1 | 2.74 | < 0.0001 | -23.4 | 3.76 |
| orbitofrontal cortex | < 0.0001 | -15.1 | 2.41 | < 0.0001 | -22.2 | 3.52 |
| parahippocampal cortex | < 0.0001 | -5.4 | 0.85 | < 0.0001 | -12.4 | 1.91 |
| hippocampus | = 0.4483 | -0.8 | 0.12 | < 0.0001 | -7.2 | 1.13 |
| thalamus | = 0.0044 | -2.9 | 0.45 | < 0.0001 | -7.4 | 1.11 |

**Supplementary Table 3**: Comparison of [^18^F]FDG-SUVR before and after PVEc

| **Region** | **% change in SUVR** | | **p-value** | | **%COV** | |
| --- | --- | --- | --- | --- | --- | --- |
|  | CN | AD | CN | AD | CN | AD |
| posterior cingulate | 42.9 | 48.5 | < 0.0001 | < 0.0001 | 8.2🡪8.9 | 10.3🡪9.2 |
| superior parietal (including the precuneus) | 73.0 | 83.0 | < 0.0001 | < 0.0001 | 7.4🡪8.7 | 10.5🡪9.3 |
| orbitofrontal cortex | 84.0 | 93.3 | < 0.0001 | < 0.0001 | 6.9🡪8.3 | 7.4🡪7.0 |
| parahippocampal cortex | 38.9 | 47.7 | < 0.0001 | < 0.0001 | 6.7🡪7.1 | 9.4🡪9.3 |
| hippocampus | 15.2 | 26.2 | < 0.0001 | < 0.0001 | 6.7🡪6.7 | 10.4🡪8.2 |
| thalamus | 67.0 | 81.8 | < 0.0001 | < 0.0001 | 9.6🡪8.3 | 10.1🡪9.6 |

CN: controls, AD: Alzheimer’s disease patients

**Supplementary Table 4**: Comparison of [^18^F]FDG-SUVR of controls and Alzheimer’s disease patients

| **Region** | **non-corrected** | | | **PVEc** | | |
| --- | --- | --- | --- | --- | --- | --- |
|  | p | T | Cohen’s d’ | p | T | Cohen’s d’ |
| posterior cingulate | < 0.0001 | 12.3 | 1.81 | < 0.0001 | 9.8 | 1.41 |
| superior parietal (including the precuneus) | < 0.0001 | 11.0 | 1.65 | < 0.0001 | 6.9 | 1.01 |
| orbitofrontal cortex | < 0.0001 | 8.1 | 1.18 | = 0.0017 | 3.2 | 0.45 |
| parahippocampal cortex | < 0.0001 | 11.7 | 1.74 | < 0.0001 | 6.5 | 0.96 |
| hippocampus | < 0.0001 | 11.3 | 1.71 | < 0.0001 | 5.0 | 0.74 |
| thalamus | < 0.0001 | 7.6 | 1.11 | = 0.0721 | 1.8 | 0.27 |

**Supplementary Figures**


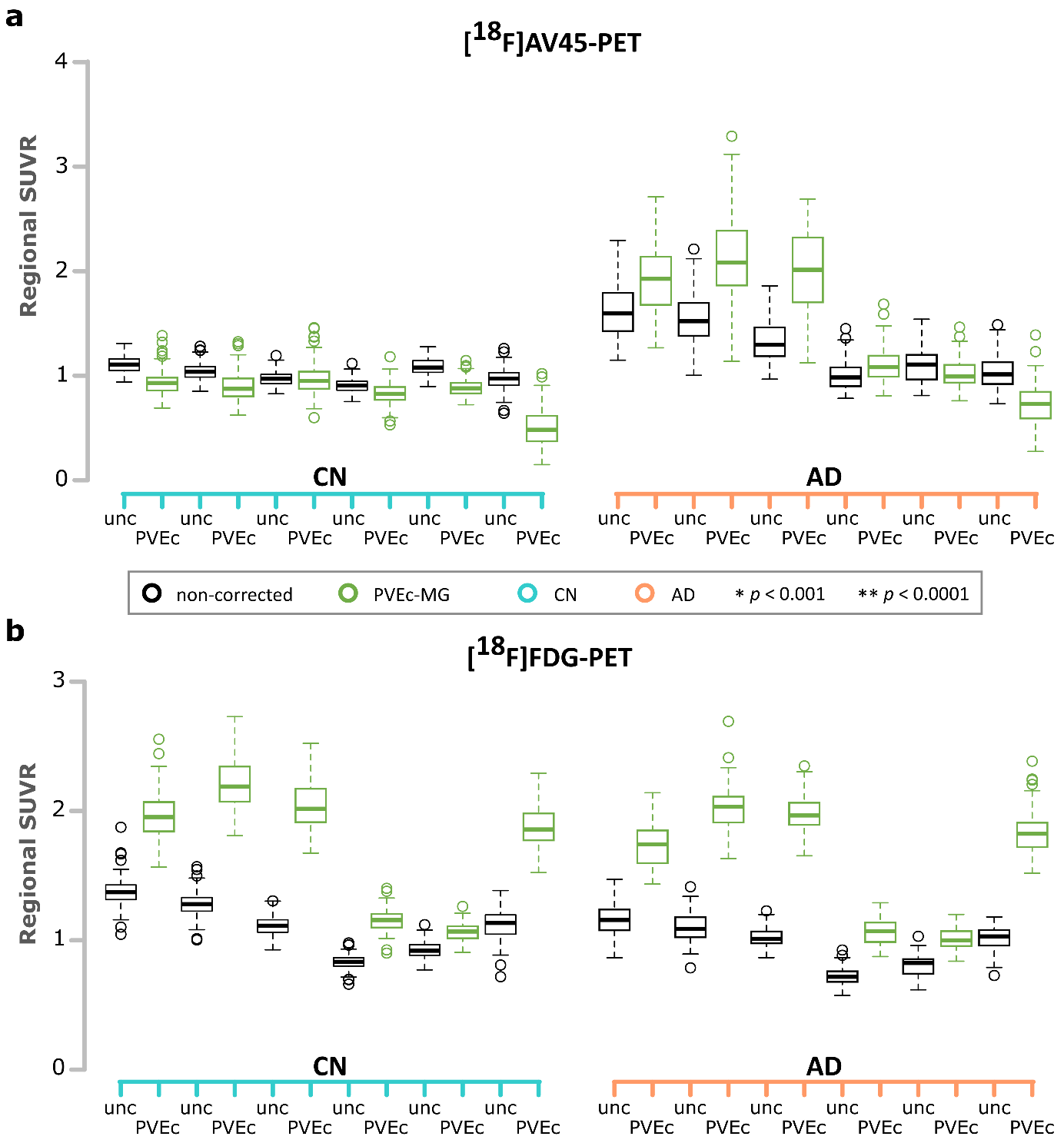


**Supplementary Figure 1.** Regional standard uptake value ratios (SUVR) for (a) [^18^F]AV45-PET and (b) [^18^F]FDG-PET. Box-plots depicting the distribution of the regional data before and after partial volume effects correction (PVEc) and further indicating the increased variability outside upper and lower quartiles after PVEc. On each boxplot, the central mark indicates the median, and the bottom and top edges of the box the upper (75th) and lower (25th) percentiles, the whiskers extend to the most extreme points of the data distribution that are not considered as outliers, while outliers are plotted beyond with a circle. From left to right the regions for each group are: the posterior cingulate, superior parietal lobe (including the precuneus), orbitofrontal cortex, parahippocampal cortex, hippocampus and thalamus.


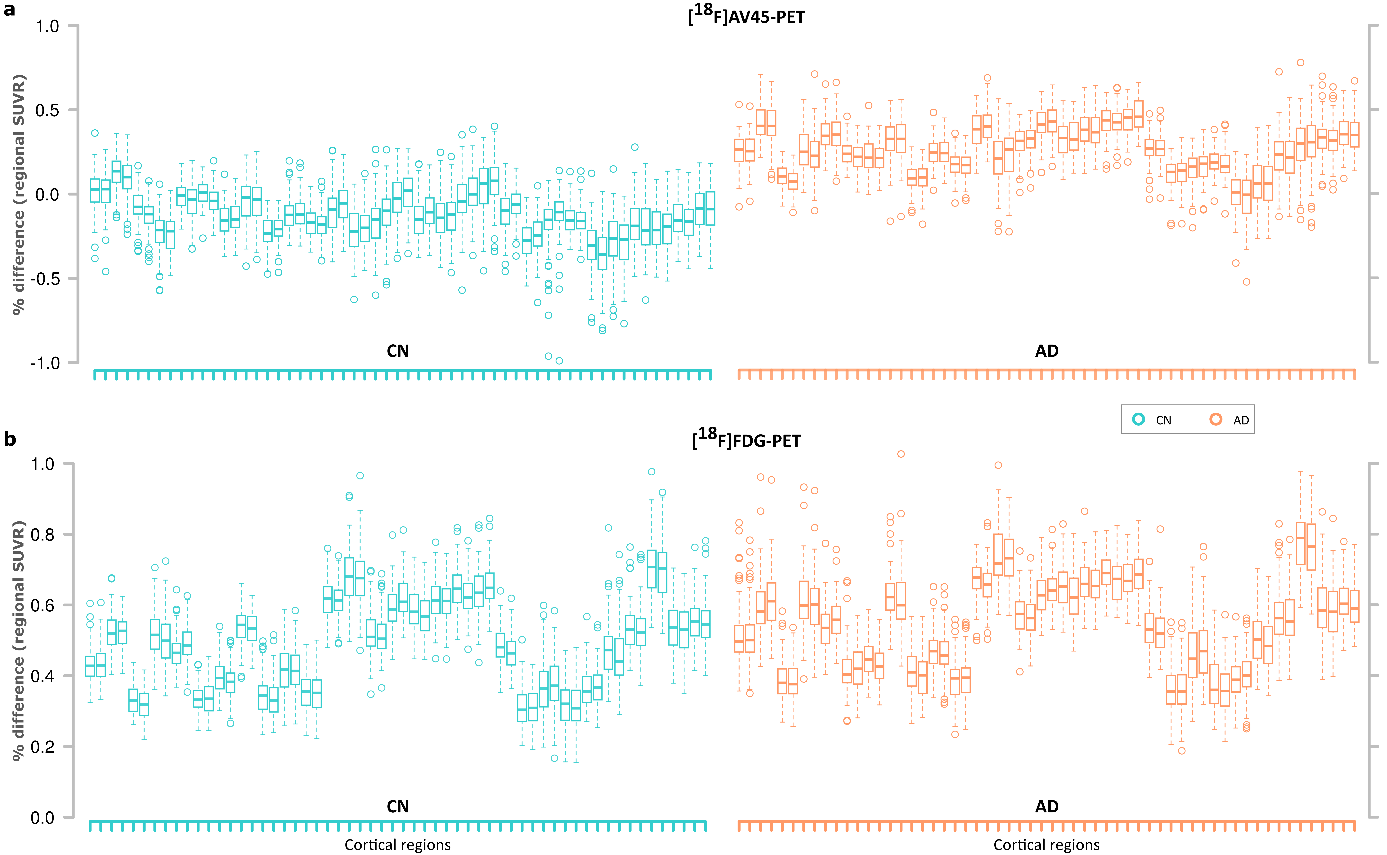


**Supplementary Figure 2.** Regional standard uptake value ratios (SUVR) for (a) [^18^F]AV45-PET and (b) [^18^F]FDG-PET. Box-plots depicting the percent differences in regional SUVRs before and after partial volume effects correction (PVEc). On each boxplot, the central mark indicates the median, and the bottom and top edges of the box the upper (75th) and lower (25th) percentiles, the whiskers extend to the most extreme points of the data distribution that are not considered as outliers, while outliers are plotted beyond with a circle. The boxplot depict all cortical brain regions included in the Hammers Maximum Probability atlas (Hammers et al., 2003).


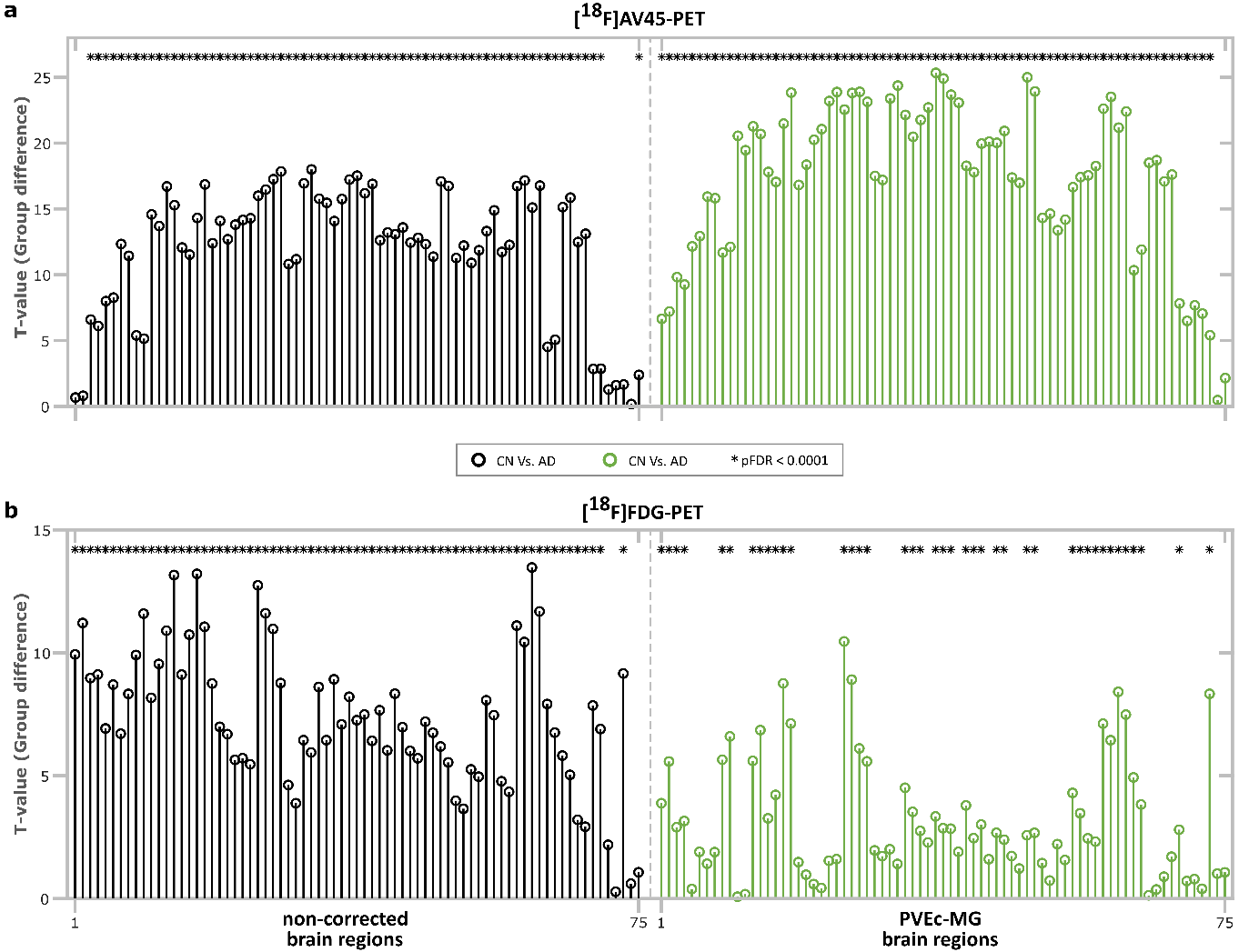


**Supplementary Figure 3.** Regional standard uptake value ratios (SUVR) for (a) [^18^F]AV45-PET and (b) [^18^F]FDG-PET. Circles depicting the T-value of the groups (CN Vs. AD) differences in regional SUVRs shown before (black circles) and after partial volume effects correction (PVEc; green circles). Asterisks mark the regions showing groups differences after correction for multiple comparisons (FDR at 0.05) across regions and correction method. Brain regions correspond to those defined in the Hammers Maximum Probability atlas (Hammers et al., 2003).


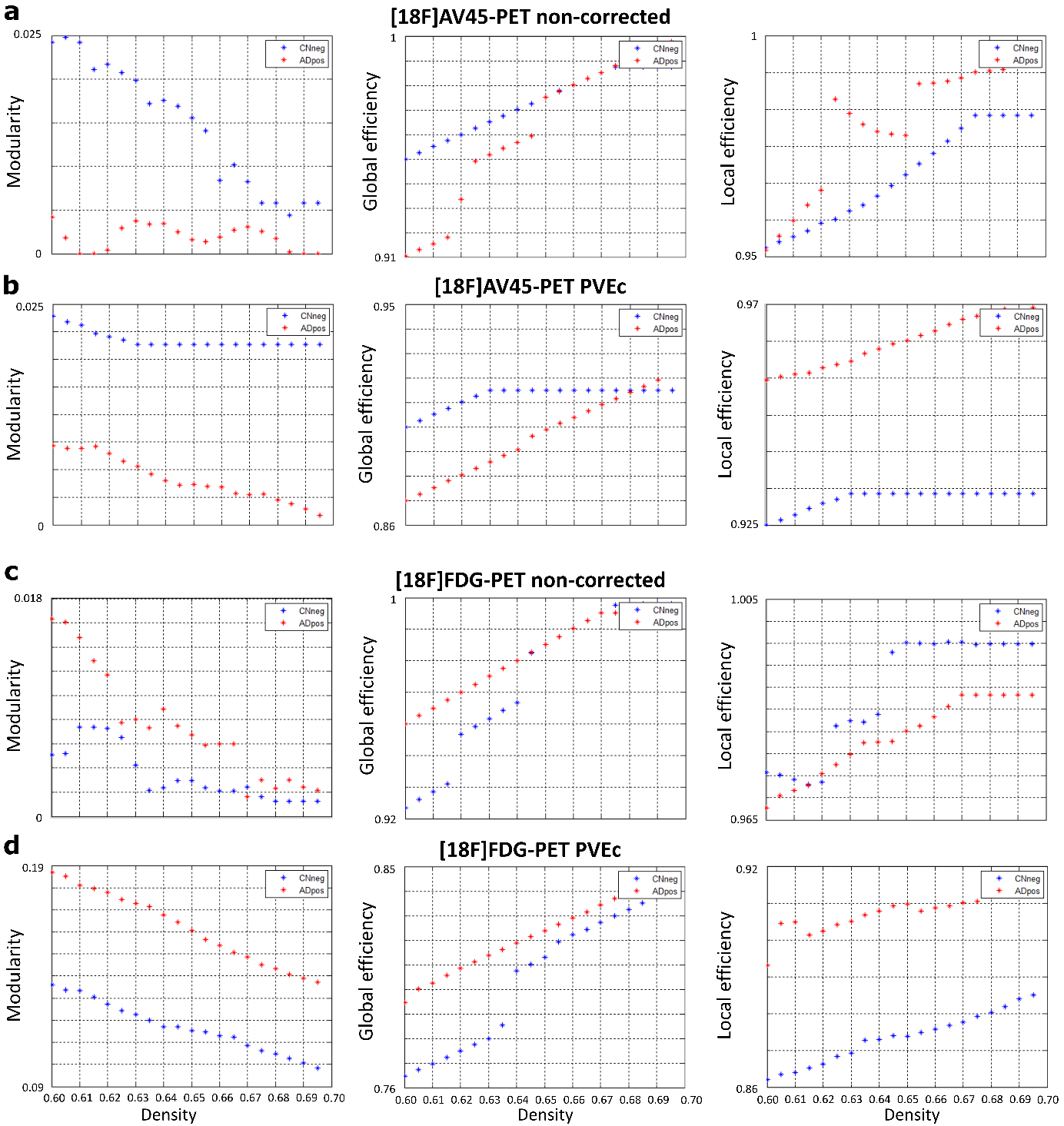


**Supplementary Figure 4.** Network metrics across densities for (a) non-corrected and (b) PVEc [^18^F]AV45-PET and (c) non-corrected and (d) PVEc [^18^F]FDG-PET data.

**Supplementary references**

Cao, Q., Shu, N., An, L., Wang, P., Sun, L., Xia, M. R., . . . He, Y. (2013). Probabilistic diffusion tractography and graph theory analysis reveal abnormal white matter structural connectivity networks in drug-naive boys with attention deficit/hyperactivity disorder. *Journal of Neuroscience, 33*(26), 10676-10687. doi: 10.1523/JNEUROSCI.4793-12.2013

Drakesmith, M., Caeyenberghs, K., Dutt, A., Lewis, G., David, A. S., & Jones, D. K. (2015). Overcoming the effects of false positives and threshold bias in graph theoretical analyses of neuroimaging data. *NeuroImage, 118*, 313-333. doi: 10.1016/j.neuroimage.2015.05.011

He, Y., Dagher, A., Chen, Z., Charil, A., Zijdenbos, A., Worsley, K., & Evans, A. (2009). Impaired small-world efficiency in structural cortical networks in multiple sclerosis associated with white matter lesion load. *Brain, 132*(Pt 12), 3366-3379. doi: 10.1093/brain/awp089

Hosseini, S. M., Hoeft, F., & Kesler, S. R. (2012). GAT: a graph-theoretical analysis toolbox for analyzing between-group differences in large-scale structural and functional brain networks. *PLoS One, 7*(7), e40709. doi: 10.1371/journal.pone.0040709

Latora, V., & Marchiori, M. (2001). Efficient behavior of small-world networks. *Phys Rev Lett, 87*(19), 198701. doi: 10.1103/PhysRevLett.87.198701

Newman, M. E. (2006). Modularity and community structure in networks. *Proc Natl Acad Sci U S A, 103*(23), 8577-8582. doi: 10.1073/pnas.0601602103

Rubinov, M., Knock, S. A., Stam, C. J., Micheloyannis, S., Harris, A. W., Williams, L. M., & Breakspear, M. (2009). Small-world properties of nonlinear brain activity in schizophrenia. *Human Brain Mapping, 30*(2), 403-416. doi: 10.1002/hbm.20517

Rubinov, M., & Sporns, O. (2010). Complex network measures of brain connectivity: uses and interpretations. *NeuroImage, 52*(3), 1059-1069. doi: 10.1016/j.neuroimage.2009.10.003

Zhang, J., Wang, J., Wu, Q., Kuang, W., Huang, X., He, Y., & Gong, Q. (2011). Disrupted brain connectivity networks in drug-naive, first-episode major depressive disorder. *Biol Psychiatry, 70*(4), 334-342. doi: 10.1016/j.biopsych.2011.05.018
